# Antiretroviral therapy optimisation in the time of COVID-19: Is it really different in North and South Africa?

**DOI:** 10.4102/sajhivmed.v21i1.1118

**Published:** 2020-07-30

**Authors:** Ahmed Cordie, Menna-t-allah El-Kotamy, Gamal Esmat

**Affiliations:** 1 Endemic Medicine Department, Cairo University Hospitals, Cairo, Egypt; 2 Egyptian Patent Office, Academy of Scientific Research and Technology, Cairo, Egypt

Dear editors, we are attentively pursuing calls for an urgent need to have global and national actions to adopt differentiated service delivery (DSD) to ensure continuity of human immunodeficiency virus (HIV) services, especially uninterrupted antiretroviral therapy (ART) supply, during the COVID-19 (Coronavirus Disease 2019) pandemic.^1^ Location modification, longer refill times and tailored packages of clinical services, including ART optimisation, are the main pillars of the transition toward DSD.^2^

However, these interventions alone are not sufficient in low- to middle-income countries (LMICs), where the lockdown and restrictions applied to international movement may affect ART supply, especially for imported medications. Medicine stock-outs are an unfortunate possibility for treatment discontinuation and the emergence of drug resistance.^3^

Following the World Health Organization (WHO) recommendations, the Egyptian guidelines set the combination of tenofovir disoproxil fumarate (TDF) and emtricitabine (FTC) plus dolutegravir (DTG) as the preferred first-line treatment regimen for adults and TDF/FTC/efavirenz (EFV) as an alternative first-line regimen.^4,5^

Egypt is reported to have very low HIV prevalence; however, it has the fastest increasing epidemic in the Middle East and North African regions.^6,7^ The Medicines Policy and Standards (PSM) system can guarantee uninterrupted ART supply when the framework cycle is properly functioning at all levels of the healthcare system. Unfortunately, LMICs usually have underdeveloped PSMs, and hence face the risk of stock-outs.^3^

During the COVID-19 crisis, the situation is expected to be more complicated; therefore, resilience is needed to enable the health system to follow the WHO treatment recommendation guidelines and keep treatment for all as a first priority,^4^ at the same time as the context is rapidly evolving. Therefore, ART included in national treatment regimens may need to be locally manufactured for the time being.

Our search revealed that FTC and EFV are not patented in Egypt, and the only patent on lamivudine (3TC) has expired. Tenofovir disoproxil fumarate, 3TC and EFV are all locally manufactured and available at an affordable price. Dolutegravir (a ViiV product), provided under voluntary license in South Africa, is part of a patent filed in Egypt that was technically rejected and is still under appeal; however, it is also provided under voluntary license. Gilead Sciences have patents on TDF/FTC in a number of countries, but not in Egypt or South Africa.^8^

Neither DTG nor the TDF/FTC 2-in-1 combination is locally manufactured in Egypt. In these critical times, local pharmaceutical companies should be encouraged to produce these medicines to avoid dependence on the originators, who do not even have patent rights in Egypt.

Currently, Egypt and many LMICs are in an extraordinary situation. These desperate times demand extraordinary measures. With respect to the WHO first-line ART recommendation for adults during the COVID-19 crisis, to ensure stable ART supply, we recommend the following:

- Antiretroviral therapy–naïve patients should start locally produced TDF + 3TC + EFV, as this stock may be less threatened than the TDF/FTC 2-in-1 combination and DTG-based regimens.
- In contradiction to what is recommended in South Africa and many sub-Saharan countries, we recommend slowing down the transition from EFV to DTG to save it for those already using it.
- In the case of stock-outs, virally suppressed patients on the TDF/FTC 2-in-1 combination-based regimen can be switched safely to locally produced TDF + 3TC, as the available evidence confirms the interchangeability between FTC and 3TC.^9^ Healthcare workers can follow ART-switching guidelines that can be applied by means of telemedicine.^10^

We should highlight that increased pill burden (four instead of two pills) is the main limitation of this recommended locally manufactured regimen. However, the priority under the current circumstances is to ensure uninterrupted ART supply.
